# Potential prevention of small for gestational age in Australia: a population-based linkage study

**DOI:** 10.1186/1471-2393-13-210

**Published:** 2013-11-19

**Authors:** Lee K Taylor, Yuen Yi Cathy Lee, Kim Lim, Judy M Simpson, Christine L Roberts, Jonathan Morris

**Affiliations:** 1 Centre for Epidemiology and Evidence, New South Wales Ministry of Health, Sydney, Australia; 2 School of Public Health, University of Sydney, Sydney, Australia; 3 Kolling Institute of Medical Research, University of Sydney, Sydney, Australia

**Keywords:** Small for gestational age, Population attributable fraction, Record linkage

## Abstract

**Background:**

Small for gestational age (SGA) infants are at increased risk of morbidity and mortality. We sought to identify risk factors associated with SGA and examined the potential for reducing the proportion of infants with SGA at a population level.

**Methods:**

Birth and hospital records were linked for births occurring in 2007–2010 in New South Wales, Australia. The analysis was stratified into three groups: preterm births, term births to non-diabetic mothers and term births to diabetic mothers. Logistic regression was used to examine the association between SGA and a range of socio-demographic and behavioural factors and health conditions, with generalised estimating equations to account for correlation among births to the same mother. Model-based population attributable fractions (PAFs) were calculated for risk factors that were considered causative and potentially modifiable.

**Results:**

Of 28,126 SGA infants, the largest group was term infants of non-diabetic mothers (88.5%), followed by term infants of diabetic mothers (6.3%) and preterm infants (5.3%). The highest PAFs were for smoking: 12.4% for preterm SGA and 10.3% for term SGA infants of non-diabetic mothers. Other risk factors for SGA that were considered modifiable included: illicit drug dependency or abuse in pregnancy in all three groups, and pregnancy hypertension and late commencement of antenatal care in term infants of non-diabetic mothers, but PAFs were less than 3%.

**Conclusions:**

There are opportunities for modest reduction of the prevalence of SGA through reduction in smoking in pregnancy, and possibly earlier commencement of antenatal care and improved management of high-risk pregnancies.

## Background

Every year more than 20 million infants are born with low birth weight (LBW) (less than 2500 grams), accounting for 15% of births globally. Infants with LBW are at increased risk of morbidity and mortality, inhibited growth and cognitive development, and poor quality of life in infancy, childhood and adult life [1]. LBW can result from prematurity, intrauterine growth retardation, or a combination of these. Small for gestational age (SGA) is preferable to LBW as a measurement of adequate fetal growth as SGA takes into account the variation in birthweights across gestational ages and infant sex. While the definition of SGA varies according to the context [2], SGA is typically defined as birthweight less than the 10th percentile of the population gestational age- and sex-specific birthweight percentiles [3].

The pathophysiological mechanisms of SGA are multifactorial, and are attributable to a complex interplay between socio-demographic, biological, medical and behavioural factors [2]. Socio-demographic factors include: Aboriginality (Aboriginal mothers) [4], minority race [5], marital status (single and unmarried mothers) [6], maternal age (younger and older mothers) [3,7] and socio-economic status (socioeconomically disadvantaged) [8]. Obstetric history factors include: nulliparity [9], previous infant with SGA [3,10], previous preterm birth [11], previous stillbirth [3], previous abortion [12], maternal history of being SGA [11], and short and long inter-pregnancy interval [13]. Chronic and pregnancy-specific medical conditions include: diabetes [3], hypertension [14], placenta abruption [3], placenta praevia [3], chronic diseases (cardiac disease [15], chronic kidney disease [3], asthma/chronic obstructive pulmonary disease [16], thyroid disorder and autoimmune disease [3]) and infections such as urinary tract infection [10]. Maternal health behaviours during pregnancy include: smoking [3], illicit drug use [17], alcohol use [18] and lack of nutritional intake [11]. Other factors include: low maternal weight gain [11], body mass index [3], inadequate antenatal care [11], assisted reproductive technology use [19], exposure to toxic substances [11], maternal work and psychosocial stress [11], parental factors [11], vigorous physical activity [10] and fetus with congenital anomaly [11].

There is little information on the risks for SGA at a population level [20-22]. A recently published population-based study from the British Columbia Perinatal Database Registry investigated a limited set of maternal risk factors for SGA (i.e., maternal age, maternal height and weight, smoking in pregnancy, hypertension and placental disorders) as potential targets of intervention to reduce socio-economic SGA inequality by calculating the population attributable fractions for each factor [22]. Other published estimates of population attributable fraction are largely limited to only smoking in pregnancy [23-25].

New South Wales (NSW) is the most populous state in Australia with approximately 7 million people, representing 32% of the Australian population [26]. In 2010 there were 96,486 births in NSW, accounting for 32% of births in Australia [27]. The NSW Government’s State Health Plan–A New Direction for NSW towards 2010 on Strengthening Primary Health and Continuing Care in the Community aims to counteract the impact of genetic, social and environmental factors which predispose infants to ill health, through promotion and implementation of early interventions that lead to the birth of healthy infants and assist them in reaching their full potential for healthy and productive lives [28]. To inform the NSW Government on priority areas for prevention activities, it is important to identify and quantify risk factors influencing SGA infants at a population level; of particular interest are potentially modifiable risk factors. The aims of this study were to identify population risk factors associated with SGA among preterm and term infants and examine the potential for reducing the proportion of infants with SGA at a population level.

## Methods

### Study population

The study population comprised 299,190 singleton live births with gestational age between 24 and 44 weeks in NSW from 2007 to 2010 for 249,429 mothers with a complete obstetric history. Mothers with a complete obstetric history were defined as those who gave birth in 2007 to 2010 with the reported number of previous pregnancies at each birth consistent with the number of linked historical birth records in the period 1994 to 2010. Restriction by gestational age was a consequence of unreliable percentile estimates for infants less than 24 or greater than 44 weeks gestation due to insufficient data. Multifetal pregnancies and stillbirths were excluded, as they carry a different set of risks compared to singleton live births.

### Data sources

Data were obtained from two linked NSW population databases: the Perinatal Data Collection (PDC) and Admitted Patient Data (APD). The PDC is a legislated population-based surveillance system covering all live births, and stillbirths of at least 20 weeks gestation or at least 400 grams birthweight. Information is recorded by either the midwife or medical practitioner providing maternity care and includes demographic, medical and obstetric information on the mother, as well as infant outcomes. The APD is a census of all admitted patient services that includes summary discharge information for every inpatient that is admitted to any NSW public or private hospital. Diagnosis and procedures for each admission are coded according to the 10th revision of the International Classification of Disease, Australian Modification and the Australian Classification of Health Intervention respectively [29].

Record linkage was carried out by the NSW Centre for Health Record Linkage (CHeReL) [30] using a best practice approach in preserving privacy [31] and the open source probabilistic record linkage software ChoiceMaker [32]. This involves a process of blocking and matching combinations of the following variables with identifying information: full name, address, sex, date of birth, country of birth, hospital code, medical record number, hospital dates of admission and discharge, hospital transferred to, hospital transferred from, and date of death. Each match was assigned a probability match weight and borderline matches were subject to clerical review [33]. Longitudinally linked birth records from 1994 to 2010 were used to obtain an obstetric history for mothers who gave birth during 2007 to 2010. Birth and hospital records were linked cross-sectionally for the hospitalisation where the woman gave birth and longitudinally for hospitalisations prior to birth. Over 99% of birth records linked to a hospital record. The CHeReL reported the quality of the record linkage for this study as less than one in 1000 false positive links and less than two in 1000 missed links. The researchers were provided with anonymised data. Ethical approval for the study was obtained from the NSW Population and Health Services Research Ethics Committee.

### Risk factors

The following information was obtained from birth records: socio-demographic factors including mother’s Aboriginality, country of birth, maternal age, antenatal care visit less than 14 weeks gestation, and socio-economic status (the Australian Bureau of Statistics Index of Economic Resources) [34] and geographic remoteness (Accessibility/Remoteness Index of Australia) [35] based on Statistical Local Area of residence; and information derived from the complete obstetric history including parity, inter-pregnancy interval, previous birth by caesarean section, previous preterm birth, previous stillbirth and previous SGA infant. The following information was obtained from birth records linked to hospital records: maternal medical conditions including diabetes (pre-existing or gestational), hypertensive disorders (chronic or gestational hypertension, preeclampsia or eclampsia), placenta abruption, placenta praevia, urinary tract infection, chronic diseases (fives years lookback) [36] (cardiac diseases, chronic kidney disease, asthma/chronic obstructive pulmonary disease (COPD), thyroid disorders and autoimmune diseases), and maternal health behaviours during pregnancy including smoking, alcohol and illicit drug dependency or abuse. Information about congenital anomalies was obtained from infant birth admission records. Only variables that are accurately reported and validated were included in the analysis [37-40].

### Outcome variable

SGA was defined as less than the 10th percentile of Australian national gestational age- and sex-specific birthweight percentiles [41]. The 10th percentile for infants of 39 weeks gestation is approximately 2500 grams birthweight regardless of infant sex. Births were stratified into preterm and term groups, and SGA and non-SGA infants were compared within each group. Birthweight is recorded to the nearest five grams. Gestational age is recorded in completed weeks of gestation determined by the best clinical estimate based on early pregnancy ultrasound or the first day of the last menstrual period.

### Analysis

Using infants who were not SGA as the comparison group, the association between each study variable and SGA was examined for preterm and term groups. Descriptive analysis was carried out to examine univariate associations. Variance inflation factors were used to assess multicollinearity between variables. Variables with *P* < 0.10 based on crude chi-squared tests were included in the initial multivariable model. Using logistic regression and backward elimination, the overall least significant variable was progressively removed from each model until only variables significant at *P* < 0.01 remained or if they were confounders (change in adjusted odds ratio [aOR] of 10% or more). Once a main effects model was determined, all eliminated variables were added back into the model independently and their statistical significance checked. All two-way biologically plausible interactions were also considered and retained if *P* < 0.01. For term analysis, infants were divided into two groups for diabetic and non-diabetic mothers, as the risks of SGA for a number of study variables were found to be modified by whether the mother had diabetes.

Once the final model was determined, generalised estimating equations (GEEs) with a logit link, an exchangeable working correlation matrix and robust standard errors were used to obtain the aORs, while accounting for correlation among births to the same mother in the study period [42]. Model-based population attributable fractions (PAFs) for risk factors that were considered causative and potentially modifiable were obtained by calculating the difference in the expected number of SGA infants between the final adjusted model and the same model with the risk factor’s regression coefficient(s) set to zero (representing removal from the population and/or effective management), as a percentage of the former [43]. Ninety-five per cent bias-corrected confidence intervals were computed via bootstrap with 10,000 replicates. The PAFs represent the greatest attainable reduction in the rate of SGA that would be observed if the population was free of the risk factor of interest. Analysis was carried out using SAS 9.3 (SAS Institute, USA) [44] and Stata SE 11.1 (Statacorp, USA) [45].

## Results

There were 308,989 mothers with at least one singleton live birth with a gestational age between 24 and 44 weeks in NSW in the period 2007 to 2010. Of these, 259,380 (83.9%) had a complete obstetric history: 28,197 (85.5%) mothers of SGA infants and 237,207 (83.8%) mothers of non-SGA infants. Incomplete obstetric history was more likely to occur in mothers with high-risk pregnancy, i.e., older maternal age (≥35 years: 37.9% vs. 20.8%), multiparous, socio-economically disadvantaged (4th–5th quintiles: 45.0% vs. 37.9%), residence in a geographically remote area (10.5% vs. 8.6%), smoking in pregnancy (18.1% vs. 11.1%) and late commencement of antenatal care (24.9% vs. 18.6%). Of the 312,047 birth records for the 308,989 mothers with a complete obstetric history, 99.1% were linked to hospital records, and 96.8% of these linked birth and hospital records had complete information for all variables of interest. The final analysis dataset comprised 299,190 singleton live births relating to 249,429 mothers: 201,456 (80.8%) mothers had one birth, 46,215 (18.5%) mothers had two births and 1758 (0.7%) mothers had three or more births.

Of all singleton live births, 15,541 (5.2%) were preterm and 283,649 (94.8%) were term. There were 28,126 SGA infants, of whom 1478 (5.3%) were preterm, 24,883 (88.5%) were term infants of non-diabetic mothers, 1765 (6.3%) were term infants of diabetic mothers. Crude results are presented separately for the three groups—preterm SGA infant, term SGA infant of diabetic mother and term SGA infant of non-diabetic mother (Additional file 1: Tables S1, Additional file 2: Table S2 and Additional file 3: Table S3). Adjusted odds ratios and population attributable fractions for the three groups are presented in Table 1.

**Table 1:** Adjusted odds ratios and population attributable fractions for SGA infants, New South Wales, 2007–2010

**Study variable**	**Preterm infant**		**Term infant, diabetic mother**		**Term infant, non-diabetic mother**	
	**Adjusted OR (95% CI)**	**Model-based PAF (95% CI)**	**Adjusted OR (95% CI)**	**Model-based PAF (95% CI)**	**Adjusted OR (95% CI)**	**Model-based PAF (95% CI)**
Country of birth, Aboriginality						
Aboriginal Australian	1.08 [0.84, 1.39]	0.36 [-0.92, 1.68]	1.08 [0.75, 1.57]	0.13 [-0.48, 0.80]	1.29 [1.20, 1.38]	0.87 [0.61, 1.12]
Non-Aboriginal Non-Australian†	1.00		1.00		1.00	
Non-Australian	1.67 [1.46, 1.90]	11.41 [8.36, 14.41]	1.92 [1.72, 2.14]	27.01 [22.74, 31.23]	1.54 [1.50, 1.59]	11.08 [10.29, 11.86]
ARIA+remoteness						
Major cities	0.88 [0.77, 1.01]		Not retained		Not retained	
Inner regional†	1.00					
Outer regional	0.77 [0.60, 0.98]					
Remote	0.36 [0.17, 0.78]					
Socio-economic group						
1st quintile (Most advantaged)†	(1st–4th quintiles vs. 5th quintile)		1.00		(1st–3rd quintiles vs. 4th, 5th quintile)	
2nd quintile			1.15 [0.97, 1.35]			
3rd quintile			1.06 [0.89, 1.26]			
4th quintile			1.23 [1.02, 1.47]		1.12 [1.08, 1.16]	
5th quintile (Most disadvantaged)	1.22 [1.07, 1.40]		1.41 [1.21, 1.64]		1.18 [1.15, 1.22]	
Inter-pregnancy interval						
6–41 months, nulliparity†	Not retained		Not retained		1.00	
<6 or ≥42 months					1.14 [1.08, 1.19]	
Number of previous pregnancies						
0	1.89 [1.62, 2.19]	26.97 [21.25, 32.52]	2.71 [2.38, 3.09]	39.82 [35.22, 44.14]	2.73 [2.62, 2.84]	36.96 [35.80, 38.13]
1†	1.00		0.66 [0.53, 0.83]		1.00	
2	0.57 [0.45, 0.70]		0.56 [0.41, 0.77]		0.71 [0.67, 0.74]	
3	(0, 2+ vs. 1)		(0,2+ vs. 1)		0.55 [0.51, 0.61]	
4+					0.47 [0.41, 0.55]	
Number of previous births by caesarean						
0†	Not retained		Not retained		1.00	
1					0.92 [0.87, 0.97]	
2					(None vs. Any)	
3+						
Number of previous preterm births						
0†	Not retained		Not retained		1.00	
1					1.68 [1.55, 1.82]	1.17 [0.95, 1.38]
2+					2.00 [1.46, 2.72]	0.11 [0.55, 0.17]
Number of stillbirths						
0	Not retained		Not retained		1.00	
1+					1.40 [1.14, 1.72]	0.12 [0.04, 0.20]
Number of previous SGA infants						
0†	1.00		1.00		1.00	
1	3.70 [3.02, 4.54]	9.81 [8.00, 11.67]	4.00 [3.28, 4.88]	7.27 [5.91, 8.73]	4.90 [4.67, 5.14]	11.16 [10.73, 11.62]
2	7.56 [5.03, 11.36]	3.11 [2.22, 4.13]	8.93 [5.52, 14.42]	1.44 [0.89, 2.06]	12.27 [10.93, 13.77]	2.24 [2.06, 2.43]
3+	–		–		16.89 [13.10, 21.78]	0.54 [0.45, 0.65]
Pre-existing/Gestational diabetes	0.60 [0.49, 0.74]		Not retained		Not retained	
Pregnancy hypertension	4.46 [3.94, 5.05]	28.80 [25.97, 31.64]	Not retained		1.17 [1.12, 1.22]	1.22 [0.84, 1.89]
Chronic hypertension	3.12 [2.34, 4.16]	2.73 [1.80, 3.78]	Not retained		1.31 [1.14, 1.50]	0.20 [0.09, 0.32]
Illicit drug use during pregnancy	1.82 [1.29, 2.57]	1.45 [0.52, 2.43]	3.25 [1.46, 7.22]	0.31 [0.04, 0.64]	2.00 [1.74, 2.29]	0.57 [0.44, 0.70]
Smoking during pregnancy	2.23 [1.91, 2.61]	12.41 [9.79, 14.99]	1.45 [1.21, 1.75]	2.51 [1.14, 3.92]	2.31 [2.22, 2.40]	10.28 [9.77, 10.83]
First antenatal care visit ≥14 weeks	Not retained		Not retained		1.05 [1.01, 1.08]	0.79 [0.20, 1.39]
Fetus with congenital anomaly	2.26 [1.86, 2.74]		Not retained		1.46 [1.35, 1.59]	

*SGA*, small-for-gestational-age, *OR*, odds ratio, *CI*, confidence interval, *PAF*, population attributable fraction, *ARIA+*, accessibility/remoteness index of Australia.

†Reference category. For dichotomised variables, the reference category is absence of variable.

In the crude analysis, the following risk factors were found to be associated with SGA infant:

▪ All three groups: overseas-born mother, increasing socio-economic disadvantage, inter-pregnancy interval (<6 or ≥42 months), nulliparity, increasing number of previous SGA infants, illicit drug dependency or abuse in pregnancy, and smoking in pregnancy; and a protective effect of previous caesarean section.

▪ Preterm SGA infant: additionally, Aboriginal mother, chronic or pregnancy hypertension, autoimmune diseases and fetus with a congenital anomaly; and protective effects of residence in a geographically remote area, two previous caesarean sections, diabetes and placenta praevia.

▪ Term SGA infant of a diabetic mother: additionally, residence in a major city and late commencement of antenatal care; and protective effects of previous preterm birth and chronic kidney disease.

▪ Term SGA infant of a non-diabetic mother: additionally, Aboriginal mother, increasing geographic remoteness of residence, younger maternal age, increasing number of previous preterm births, chronic or pregnancy hypertension, placental abruption, urinary tract infection, asthma/COPD, alcohol use in pregnancy and late commencement of antenatal care; and there was a U-shaped association with number of previous pregnancies.

After multivariable adjustment, the highest risk of a SGA infant for all three groups was associated with increasing number of previous SGA infants, with a dose–response relationship that was strongest for term SGA infants of non-diabetic mothers; while illicit drug dependency or abuse in pregnancy, smoking in pregnancy, nulliparity and overseas-born mother remained moderately strong risk factors with aORs of 1.5 to 3.2 across the groups. Socio-economic disadvantage was also retained in all three groups; while the effect was relatively small with aORs of less than 1.5, a clear pattern of increasing risk of SGA with increasing socio-economic disadvantage was observed for term SGA infants of non-diabetic mothers. In addition, for preterm SGA infants, pregnancy hypertension and fetus with a congenital anomaly remained as strong risk factors, while maternal diabetes provided a significant protective effect. For non-diabetic mothers, Aboriginal mothers retained a higher risk for a term SGA infant, as did mothers with previous preterm births, chronic or pregnancy hypertension, placental abruption and fetus with a congenital anomaly; previous caesarean section retained a slight protective effect.

There was substantial variation in PAFs for SGA across the three infant groups. Among preterm infants, the greatest risk for SGA was associated with pregnancy hypertension (PAF 28.8%), followed by smoking in pregnancy (PAF 12.4%) and previous SGA (PAFs 3.1–9.8%), with chronic hypertension and illicit drug dependency or abuse in pregnancy having modest PAFs of 1.5–3%. Among term infants of diabetic mothers, a similar pattern was observed, but to a much lesser extent (e.g., previous SGA: PAF 1.4–7.3% and smoking in pregnancy: PAF 2.5%). Among term infants of non-diabetic mothers, SGA was highly attributable to previous SGA infant (PAFs 0.5–11.2%) and smoking in pregnancy (PAF 10.3%). Previous preterm birth or stillbirth, chronic hypertension, placenta abruption, placenta praevia, illicit drug dependency or abuse in pregnancy and late commencement of antenatal care each contributed a PAF of less than 1.5%.

## Discussion

We examined risk factors for SGA and estimated PAFs for potentially modifiable factors among three groups of liveborn infants of at least 24 weeks gestation in NSW. Of the 28,126 SGA infants in the study, the largest group (88.5%) was term infants of non-diabetic mothers, while 6.3% were term infants of diabetic mothers and 5.3% were preterm infants.

After multivariable adjustment, we found several risk factors that were consistently associated with SGA for all three groups of infants examined. These factors include: overseas-born mothers, nulliparity, increasing socio-economic disadvantage, increasing number of previous SGA infants, and smoking and illicit drug dependency or abuse in pregnancy. Of these, smoking and illicit drug dependency or abuse in pregnancy are potentially modifiable. The PAFs for smoking in pregnancy were 12.4% for preterm SGA and 10.3% for term SGA infant of a non-diabetic mother, reflecting the relatively high prevalence of smoking as a risk factor among pregnant mothers, and 2.5% for term SGA infant of a diabetic mother, reflecting the propensity for diabetic mothers to have large infants. The PAFs for illicit drug dependency or abuse in pregnancy were low and ranged from 0.3% to 1.5% among the three infant groups. The effect of illicit drug dependency or abuse in pregnancy on SGA is likely to be under-estimated as it is only recorded on the hospital data where there is a history of dependency or abuse and this affects care during the hospital admission.

For term infants of non-diabetic mothers, the largest population of SGA infants, the remaining risk factors for SGA that were considered modifiable included chronic or pregnancy hypertension and late commencement of antenatal care at 14 weeks gestation or more, though the PAFs for these were low at 1.2% and 0.2% for pregnancy and chronic hypertension respectively, and 0.8% for late commencement of antenatal care.

A history of previous preterm birth, stillbirth or SGA infant was associated with term SGA infant of both diabetic and non-diabetic mothers in the current pregnancy. A history of previous SGA infant was associated with preterm SGA infant in the current pregnancy. While past medical or obstetric history is not modifiable for the current pregnancy, the PAFs for term infants of non-diabetic mothers with a history of one previous SGA infant was 11.2% and for preterm infants was 9.8%, highlighting the importance of careful monitoring of these mothers in the current pregnancy.

We found some factors were protective against having a SGA infant in the current pregnancy. Previous pregnancies and previous caesarean section in a term infant of a non-diabetic mother were slightly protective against SGA in the current pregnancy. This may be related to closer monitoring of these mothers leading to delivery of infants who are failing to grow or otherwise at risk before the fetus becomes seriously growth restricted. Pre-existing and gestational diabetes is associated with large for gestational age infants and exhibited a protective effect on SGA in preterm infants. We also found that residence in an outer regional or remote area was protective of SGA, though only in preterm infants. In NSW, high risk pregnancies are referred to a centre with an appropriate level of care prior to birth, including referral to an interstate centre for mothers resident close to interstate borders. It is likely that, for mothers living in more geographically remote areas, higher risk preterm babies were referred to and born in interstate centres and their information was not available for this study. The strength of this study is the use of longitudinally linked population-based data allowing validation of parity of individual mothers. This ensured completeness of the obstetric histories and improved the accuracy of the selected sample [46]. An important contribution of this study is the calculation of the PAFs, which represent the potential reduction in SGA births that is achievable if the effects of the risk factors were eliminated in the population.

About 16% of mothers were excluded from the study due to an incomplete obstetric history, and incomplete obstetric history was more likely to occur in mothers with a high risk pregnancy. The adjusted odds ratios and population attributable fractions may therefore be underestimated. The patterns of characteristics for mothers with a complete or incomplete obstetric history were similar between the SGA and non-SGA groups (data not shown), suggesting that any bias would not be large. We were unable to adjust for some risk factors for SGA infant because the relevant information is not routinely collected, such as previous abortions, assisted reproductive technology use and maternal nutritional status. The persistent association between SGA and socio-economic disadvantage, and between SGA infants born to Aboriginal mothers, after multivariable adjustment, suggests the existence of some unmeasured risk factors for which socio-economic status and Aboriginality are proxies. We were also unable to identify which infants came from small families and were genetically destined to be small rather than being SGA. Birth weight is also related to mother’s country of birth; for example, babies of mothers born in Asian countries tend to be smaller and babies of mothers born in Polynesian countries tend to be larger than babies of Australian born mothers [47]. We used Australian birth weight standards, which cover the entire birth population and are likely to result in some misclassification of SGA and non-SGA at an individual level.

When considering the health of populations, the PAF is important because it takes into account both the level of individual risk and the prevalence of the risk factor in the population. We suggest that the greatest potential for prevention of SGA in the NSW maternity population lies in reducing smoking in pregnancy. A range of interventions have been shown to be effective in reducing smoking rates in mothers [48]. Smaller gains in preventing SGA could be achieved through reduction in illicit drug dependency and abuse in pregnancy, improved management of hypertension and targeted education to mothers about improving antenatal care in the first trimester, adopting healthy lifestyles and optimising care for medical conditions. There may be potential for reducing the rates of SGA infants among mothers with a history of previous preterm birth, stillbirth or SGA infant through closer monitoring of these high-risk pregnancies. Further research into the underlying biological mechanisms for SGA may reveal further potential for prevention.

## Conclusions

SGA is associated with a range of demographic, pregnancy history and behavioural factors, and maternal medical conditions. There are opportunities for modest reduction of the prevalence of SGA through reduction in smoking in pregnancy, and possibly earlier commencement of antenatal care and improved management of high-risk pregnancies.

## Competing interests

The authors declare that they have no competing interests.

## Authors’ contributions

LKT conceived the study. All authors contributed to study design, analysis planning and interpretation of data. LKT coordinated the study, provided clinical expertise and drafted the manuscript. YYL was involved in data linkage and preparation, statistical analysis and helped to draft the manuscript. KL assisted in statistical analysis and contributed to the acquisition of data. JS provided statistical expertise and a critical review of the manuscript. CLR and JM provided clinical expertise and a critical review of the manuscript. All authors have read and approved the final manuscript.

## Pre-publication history

The pre-publication history for this paper can be accessed here:

http://www.biomedcentral.com/1471-2393/13/210/prepub

## Supplementary Material

Additional file 1: Table S1 Characteristics of SGA and non-SGA preterm infants, New South Wales, 2007–2010. Click here for file

Additional file 2: Table S2 Characteristics of SGA and non-SGA term infants of diabetic mothers, New South Wales, 2007–2010. Click here for file

Additional file 3: Table S3 Characteristics of SGA and non-SGA term infants of non-diabetic mothers, New South Wales, 2007–2010. Click here for file
