# Supplementary material for: Potential prevention of small for gestational age in Australia: a population-based linkage study
Source: BMC Pregnancy Childbirth. 2013 Nov 19;13:210. doi: 10.1186/1471-2393-13-210 (PMC3835866; doi:10.1186/1471-2393-13-210)
Supplement: Additional file 1: Table S1 — Characteristics of SGA and non-SGA preterm infants, New South Wales, 2007–2010. [file 1471-2393-13-210-S1.docx]

**Supplementary Table 1 – Characteristics of SGA and non-SGA preterm infants, New South Wales, 2007–2010**

| Study variable | Preterm (<37 gestation weeks) | | |
| --- | --- | --- | --- |
|  | SGA  (*N* = 1,478) | Non-SGA  (*N* = 14,063) | Crude OR  (95% CI) |
|  | *n (%)* | *n (%)* |  |
| Country of birth, Aboriginality |  |  |  |
| Aboriginal Australian | 94 (6.4) | 711 (5.1) | 1.42 [1.13,1.78] |
| Non-Aboriginal Non-Australian† | 898 (60.8) | 9,614 (68.4) | 1.00 |
| Non-Australian | 486 (32.9) | 3,738 (26.6) | 1.40 [1.24,1.57] |
| ARIA+ remoteness |  |  |  |
| Major cities | 968 (65.5) | 9,242 (65.7) | 0.95 [0.84,1.07] |
| Inner regional† | 401 (27.1) | 3,612 (25.7) | 1.00 |
| Outer regional | 102 (6.9) | 1,064 (7.6) | 0.87 [0.69,1.09] |
| Remote | 7 (0.5) | 145 (1.0) | 0.42 [0.20,0.90] |
| Maternal age |  |  |  |
| <20 years | 79 (5.4) | 731 (5.2) | 0.95 [0.74,1.23] |
| 20-24 years | 273 (18.5) | 2,172 (15.4) | 1.13 [0.96,1.33] |
| 25-29 years† | 420 (28.4) | 3,770 (26.8) | 1.00 |
| 30-34 years | 399 (27.0) | 4,341 (30.9) | 0.82 [0.71,0.95] |
| 35-39 years | 237 (16.0) | 2,505 (17.8) | 0.85 [0.72,1.00] |
| ≥40 years | 70 (4.7) | 544 (3.9) | 1.15 [0.88,1.51] |
| Socio-economic group |  |  |  |
| 1st quintile (Most advantaged)† | 260 (17.6) | 2,845 (20.2) | 1.00 |
| 2nd quintile | 274 (18.5) | 2,838 (20.2) | 1.06 [0.88,1.26] |
| 3rd quintile | 296 (20.0) | 2,815 (20.0) | 1.15 [0.96,1.37] |
| 4th quintile | 268 (18.1) | 2,475 (17.6) | 1.18 [0.98,1.41] |
| 5th quintile (Most disadvantaged) | 380 (25.7) | 3,090 (22.0) | 1.34 [1.14,1.59] |
| Inter-pregnancy interval |  |  |  |
| 6–41 months, nulliparity† | 1,275 (86.3) | 11,917 (84.7) | 1.00 |
| <6 or ≥42 months | 203 (13.7) | 2,146 (15.3) | 1.28 [1.07,1.54] |
| Number of previous pregnancies |  |  |  |
| 0 | 943 (63.8) | 7,405 (52.7) | 1.50 [1.32,1.70] |
| 1† | 339 (22.9) | 3,996 (28.4) | 1.00 |
| 2 | 108 (7.3) | 1,697 (12.1) | 0.75 [0.60,0.93] |
| 3 | 59 (4.0) | 644 (4.6) | 1.08 [0.81,1.44] |
| 4+ | 29 (2.0) | 321 (2.3) | 1.06 [0.71,1.60] |
| Number of previous births by caesarean |  |  |  |
| 0† | 1,289 (87.2) | 11,825 (84.1) | 1.00 |
| 1 | 157 (10.6) | 1,671 (11.9) | 0.85 [0.71,1.01] |
| 2+ | 32 (2.2) | 567 (4.0) | 0.51 [0.35,0.73] |
| Number of previous preterm births |  |  |  |
| 0† | 1,312 (88.8) | 12,206 (86.8) | 1.00 |
| 1 | 150 (10.2) | 1,601 (11.4) | 0.87 [0.73,1.03] |
| 2+ | 16 (1.1) | 256 (1.8) | 0.60 [0.36,1.01] |
| Number of stillbirths |  |  |  |
| 0 | 1,452 (98.2) | 13,816 (98.2) | 1.00 |
| 1+ | 26 (1.8) | 247 (1.8) | 1.00 [0.67,1.51] |
| Number of previous SGA infants |  |  |  |
| 0† | 1,207 (81.7) | 12,810 (91.1) | 1.00 |
| 1 | 215 (14.6) | 1076 (7.7) | 2.06 [1.75,2.42] |
| 2+ | 56 (3.7) | 177 (1.3) | 3.26 [2.37,4.48] |
| Pre-existing/Gestational diabetes | 114 (7.7) | 1,487 (9.6) | 0.71 [0.59,0.87] |
| Pregnancy hypertension | 597 (40.4) | 2,150 (15.3) | 3.73 [3.33,4.18] |
| Chronic hypertension | 64 (4.3) | 376 (2.7) | 1.66 [1.27,2.17] |
| Placenta abruption | 51 (3.5) | 484 (3.4) | 1.00 [0.74,1.34] |
| Placenta praevia | 47 (3.2) | 723 (5.1) | 0.60 [0.44,0.82] |
| Urinary tract infection | 30 (2.0) | 314 (2.2) | 0.91 [0.62,1.34] |
| Cardiac disease | 29 (2.0) | 259 (1.8) | 1.08 [0.73,1.59] |
| Chronic kidney disease | 24 (1.6) | 238 (1.7) | 0.91 [0.60,1.39] |
| Asthma/Chronic obstructive pulmonary disease | 38 (2.6) | 379 (2.7) | 0.95 [0.68,1.34] |
| Thyroid disorders | 11 (0.7) | 100 (0.7) | 1.05 [0.56,1.97] |
| Autoimmune diseases | 29 (2.0) | 185 (1.3) | 1.51 [1.02,2.24] |
| Alcohol use during pregnancy | 6 (0.4) | 30 (0.2) | 1.92 [0.80,4.58] |
| Illicit drug use during pregnancy | 57 (3.9) | 247 (1.8) | 2.21 [1.64,2.98] |
| Smoking during pregnancy | 380 (25.7) | 2,271 (16.2) | 1.79 [1.57,2.02] |
| First antenatal care visit ≥14 weeks | 1,141 (77.2) | 11,129 (79.1) | 1.11 [0.98,1.27] |
| Fetus with congenital anomaly | 170 (11.5) | 804 (5.7) | 2.14 [1.80,2.55] |
| SGA: small-for-gestational-age, OR: odds ratio, CI: confidence interval, | | | |
| ARIA+: Accessibility/Remoteness Index of Australia |  |  |  |
| † Reference category. For dichotomised variables, the reference category is absence of variable. | | | |
