# Supplementary material for: Potential prevention of small for gestational age in Australia: a population-based linkage study
Source: BMC Pregnancy Childbirth. 2013 Nov 19;13:210. doi: 10.1186/1471-2393-13-210 (PMC3835866; doi:10.1186/1471-2393-13-210)
Supplement: Additional file 2: Table S2 — Characteristics of SGA and non-SGA term infants of diabetic mothers, New South Wales, 2007–2010. [file 1471-2393-13-210-S2.docx]

Supplementary Table 2 – Characteristics of SGA and non-SGA term infants of diabetic mothers, New South Wales, 2007–2010

| Study variable | Term infant, diabetic mother | | |
| --- | --- | --- | --- |
|  | SGA  (*N* = 1,765) | Non-SGA  (*N* = 15,527) | Crude OR  (95% CI) |
|  | *n (%)* | *n (%)* |  |
| Country of birth, Aboriginality |  |  |  |
| Aboriginal Australian | 35 (2.0) | 344 (2.2) | 1.35 [0.94,1.92] |
| Non-Aboriginal Non-Australian† | 628 (35.6) | 8,408 (54.2) | 1.00 |
| Non-Australian | 1,102 (62.4) | 6,775 (43.6) | 2.17 [1.96,2.41] |
| ARIA+ Remoteness |  |  |  |
| Major cities | 1,448 (82.0) | 11,486 (74.0) | 1.69 [1.46,1.94] |
| Inner regional† | 240 (13.6) | 3,223 (20.8) | 1.00 |
| Outer regional | 71 (4.0) | 747 (4.8) | 1.27 [0.96,1.68] |
| Remote | 6 (0.3) | 71 (0.4) | 1.14 [0.49,2.66] |
| Maternal age |  |  |  |
| <20 years | 23 (1.3) | 186 (1.2) | 0.96 [0.62,1.48] |
| 20-24 years | 131 (7.4) | 1,227 (7.9) | 0.82 [0.67,1.00] |
| 25-29 years† | 485 (27.5) | 3,723 (24.0) | 1.00 |
| 30-34 years | 628 (35.6) | 5,503 (35.4) | 0.87 [0.77,0.99] |
| 35-39 years | 399 (22.6) | 3,921 (25.3) | 0.78 [0.68,0.89] |
| ≥40 years | 99 (5.6) | 967 (6.2) | 0.78 [0.62,0.98] |
| Socio-economic group |  |  |  |
| 1st quintile (Most advantaged)† | 301 (17.1) | 3,127 (20.1) | 1.00 |
| 2nd quintile | 366 (20.7) | 3,194 (20.6) | 1.19 [1.01,1.40] |
| 3rd quintile | 278 (15.8) | 3,015 (19.4) | 0.95 [0.80,1.13] |
| 4th quintile | 252 (14.3) | 2,333 (15.0) | 1.13 [0.94,1.35] |
| 5th quintile (Most disadvantaged) | 568 (32.2) | 3,858 (24.9) | 1.52 [1.31,1.77] |
| Inter-pregnancy interval |  |  |  |
| 6–41 months, nulliparity† | 1,544 (87.5) | 12,841 (82.7) | 1.00 |
| <6 or ≥42 months | 221 (12.5) | 2,686 (17.3) | 1.29 [1.08,1.53] |
| Number of previous pregnancies |  |  |  |
| 0 | 1,189 (67.4) | 7,258 (46.7) | 2.16 [1.92,2.42] |
| 1† | 399 (22.6) | 5,270 (33.9) | 1.00 |
| 2 | 119 (6.7) | 2,063 (13.3) | 0.77 [0.62,0.94] |
| 3 | 52 (3.0) | 660 (4.3) | 1.03 [0.76,1.40] |
| 4+ | 6 (0.3) | 276 (1.8) | 0.28 [0.12,0.65] |
| Number of previous births by caesarean |  |  |  |
| 0† | 1,614 (91.4) | 12,724 (82.0) | 1.00 |
| 1 | 128 (7.3) | 2,182 (14.1) | 0.47 [0.39,0.56] |
| 2+ | 23 (1.3) | 621 (4.0) | 0.29 [0.19,0.44] |
| Number of previous preterm births |  |  |  |
| 0† | 1,704 (96.5) | 14,827 (95.5) | 1.00 |
| 1+ | 61 (3.5) | 700 (4.5) | 0.76 [0.58,0.99] |
| Number of stillbirths |  |  |  |
| 0 | 1,747 (99.0) | 15,370 (99.0) | 1.00 |
| 1+ | 18 (1.0) | 157 (1.0) | 1.02 [0.62,1.66] |
| Number of previous SGA infants |  |  |  |
| 0† | 1,553 (88.0) | 14,593 (94.0) | 1.00 |
| 1 | 182 (10.3) | 853 (5.5) | 1.89 [1.58,2.25] |
| 2+ | 30 (1.7) | 78 (0.5) | 3.40 [2.22,5.19] |
| Pregnancy hypertension | 206 (11.7) | 1,738 (11.2) | 1.06 [0.91,1.23] |
| Chronic hypertension | 35 (2.0) | 408 (2.6) | 0.75 [0.53,1.06] |
| Placenta abruption | 6 (0.3) | 37 (0.2) | 1.44 [0.61,3.38] |
| Placenta praevia | 17 (1.0) | 208 (1.3) | 0.73 [0.45,1.19] |
| Urinary tract infection | 12 (0.7) | 166 (1.1) | 0.91 [0.62,1.34] |
| Cardiac disease | 11 (0.6) | 226 (1.5) | 0.64 [0.36,1.15] |
| Chronic kidney disease | 15 (0.9) | 112 (0.7) | 0.42 [0.22,0.80] |
| Asthma/Chronic obstructive pulmonary disease | 30 (1.7) | 282 (1.8) | 1.19 [0.70,2.05] |
| Thyroid disorders | 16 (0.9) | 137 (0.9) | 0.93 [0.63,1.37] |
| Autoimmune diseases | 11 (0.6) | 119 (0.8) | 1.01 [0.60,1.70] |
| Alcohol use during pregnancy | 1 (0.1) | 5 (0.0) | 0.81 [0.44,1.49] |
| Illicit drug use during pregnancy | 9 (0.5) | 25 (0.2) | 1.75 [1.48,6.80] |
| Smoking during pregnancy | 162 (9.2) | 1,204 (7.8) | 3.18 [1.48,6.80] |
| First antenatal care visit ≥14 weeks | 1,389 (78.7) | 12,355 (79.6) | 1.20 [1.01,1.42] |
| Fetus with congenital anomaly | 40 (2.3) | 332 (2.1) | 1.07 [0.77,1.48] |
| SGA: small-for-gestational-age, OR: odds ratio, CI: confidence interval, | | | |
| ARIA+: Accessibility/Remoteness Index of Australia | |  |  |
| † Reference category. For dichotomised variables, the reference category is absence of variable. | | | |
