# Supplementary material for: Potential prevention of small for gestational age in Australia: a population-based linkage study
Source: BMC Pregnancy Childbirth. 2013 Nov 19;13:210. doi: 10.1186/1471-2393-13-210 (PMC3835866; doi:10.1186/1471-2393-13-210)
Supplement: Additional file 3: Table S3 — Characteristics of SGA and non-SGA term infants of non-diabetic mothers, New South Wales, 2007–2010. [file 1471-2393-13-210-S3.docx]

**Supplementary Table 3 – Characteristics of SGA and non-SGA term infants of non-diabetic mothers, New South Wales, 2007–2010**

| Study variable | Term infant, non-diabetic mother | | |
| --- | --- | --- | --- |
|  | SGA  (*N* = 24,883) | Non-SGA  (*N* = 241,474) | Crude OR  (95% CI) |
|  | *n (%)* | *n (%)* |  |
| Country of birth, Aboriginality |  |  |  |
| Aboriginal Australian | 1,211 (4.9) | 6,617 (2.7) | 2.09 [1.96,2.24] |
| Non-Aboriginal Non-Australian† | 14,883 (59.8) | 169,582 (70.2) | 1.00 |
| Non-Australian | 8,789 (35.3) | 65,275 (27.0) | 1.54 [1.50,1.58] |
| ARIA+ remoteness | 16,903 (67.9) | 159,931 (66.2) | 1.13 [1.09,1.17] |
| Major cities |  |  |  |
| Inner regional† | 5,636 (22.7) | 60,177 (24.9) | 1.00 |
| Outer regional | 2,076 (8.3) | 19,322 (8.0) | 1.14 [1.08,1.20] |
| Remote | 199 (0.8) | 1,586 (0.7) | 1.35 [1.15,1.57] |
| Very remote | 69 (0.3) | 458 (0.2) | 1.59 [1.22,2.06] |
| Maternal age | 1,487 (6.0) | 9,343 (3.9) | 1.42 [1.34,1.51] |
| <20 years |  |  |  |
| 20-24 years | 4,577 (18.4) | 35,742 (14.8) | 1.15 [1.11,1.19] |
| 25-29 years† | 7,590 (30.5) | 68,353 (28.3) | 1.00 |
| 30-34 years | 7,124 (28.6) | 78,587 (32.5) | 0.81 [0.79,0.84] |
| 35-39 years | 3,483 (14.0) | 42,382 (17.6) | 0.73 [0.70,0.77] |
| ≥40 years | 622 (2.5) | 7,067 (2.9) | 0.78 [0.71,0.85] |
| Socio-economic group |  |  |  |
| 1st quintile (Most advantaged)† | 4,679 (18.8) | 53,422 (22.1) | 1.00 |
| 2nd quintile | 4,917 (19.8) | 51,171 (21.2) | 1.10 [1.05,1.14] |
| 3rd quintile | 4,447 (17.9) | 45,880 (19.0) | 1.11 [1.06,1.16] |
| 4th quintile | 4,527 (18.2) | 41,697 (17.3) | 1.24 [1.19,1.29] |
| 5th quintile (Most disadvantaged) | 6,313 (25.4) | 49,304 (20.4) | 1.46 [1.40,1.52] |
| Inter-pregnancy interval |  |  |  |
| 6–41 months, nulliparity† | 22,050 (88.6) | 210,389 (87.1) | 1.00 |
| <6 or ≥42 months | 2,833 (11.4) | 31,085 (12.9) | 1.31 [1.25,1.37] |
| Number of previous pregnancies |  |  |  |
| 0 | 15,300 (61.5) | 111,427 (46.1) | 1.87 [1.81,1.92] |
| 1† | 6,109 (24.6) | 83,812 (34.7) | 1.00 |
| 2 | 2,330 (9.4) | 33,350 (13.8) | 0.95 [0.90,0.99] |
| 3 | 762 (3.1) | 9,429 (3.9) | 1.08 [1.00,1.17] |
| 4+ | 382 (1.5) | 3,456 (1.4) | 1.49 [1.33,1.66] |
| Number of previous births by caesarean |  |  |  |
| 0† | 22,625 (90.9) | 207,359 (85.9) | 1.00 |
| 1 | 1,847 (7.4) | 27,258 (11.3) | 0.62 [0.59,0.65] |
| 2 | 337 (1.4) | 5,868 (2.4) | 0.52 [0.47,0.58] |
| 3+ | 74 (0.3) | 102 (0.4) | 0.66 [0.52,0.83] |
| Number of previous preterm births |  |  |  |
| 0† | 23,960 (96.3) | 234,357 (97.1) | 1.00 |
| 1 | 852 (3.4) | 6,721 (2.8) | 1.23 [1.14,1.32] |
| 2+ | 71 (0.2) | 396 (0.2) | 1.73 [1.34,2.24] |
| Number of stillbirths |  |  |  |
| 0 | 24,765 (99.5) | 240,119 (99.4) | 1.00 |
| 1+ | 118 (0.5) | 1,355 (0.6) | 0.84 [0.69,1.02] |
| Number of previous SGA infants |  |  |  |
| 0† | 20,426 (82.1) | 225,449 (93.4) | 1.00 |
| 1 | 3,666 (14.7) | 14,624 (6.1) | 2.41 [2.31,2.52] |
| 2 | 640 (2.6) | 1,198 (0.5) | 5.42 [4.92,6.00] |
| 3+ | 151 (0.6) | 203 (0.1) | 7.70 [6.19,9.58] |
| Pregnancy hypertension | 2,422 (9.7) | 20,690 (8.6) | 1.16 [1.11,1.22] |
| Chronic hypertension | 246 (1.0) | 1,913 (0.8) | 1.24 [1.09,1.42] |
| Placenta abruption | 102 (0.4) | 552 (0.2) | 1.78 [1.44,2.20] |
| Placenta praevia | 205 (0.8) | 2,176 (0.9) | 0.92 [0.80,1.06] |
| Urinary tract infection | 281 (1.1) | 2,335 (1.0) | 1.15 [1.02,1.31] |
| Cardiac disease | 184 (0.7) | 1,676 (0.7) | 1.07 [0.92,1.25] |
| Chronic kidney disease | 181 (0.7) | 1,588 (0.7) | 1.08 [0.92,1.27] |
| Asthma/Chronic obstructive pulmonary disease | 483 (1.9) | 4,031 (1.7) | 1.16 [1.05,1.28] |
| Thyroid disorders | 108 (0.4) | 1,093 (0.5) | 0.96 [0.78,1.17] |
| Autoimmune diseases | 188 (0.8) | 1,860 (0.8) | 0.97 [0.83,1.13] |
| Alcohol use during pregnancy | 44 (0.2) | 135 (0.1) | 2.96 [2.09,4.17] |
| Illicit drug use during pregnancy | 376 (1.5) | 925 (0.4) | 3.85 [3.40,4.35] |
| Smoking during pregnancy | 5,184 (20.8) | 23,616 (9.8) | 2.39 [2.31,2.47] |
| First antenatal care visit ≥14 weeks | 19,659 (79.0) | 197,548 (81.8) | 1.18 [1.14,1.22] |
| Fetus with congenital anomaly | 730 (2.9) | 4,843 (2.0) | 1.48 [1.37,1.60] |
| SGA: small-for-gestational-age, OR: odds ratio, CI: confidence interval, | | | |
| ARIA+: Accessibility/Remoteness Index of Australia | |  |  |
| † Reference category. For dichotomised variables, the reference category is absence of variable. | | | |
